# Mung Bean Seed Coat Extract Promotes Diabetic Wound Healing in High-Glucose-Exposed HaCaT Keratinocytes

**DOI:** 10.3390/antiox15091119

**Published:** 2026-09-04

**Authors:** Sineenad Teerapatpaisan, Alisa Naladta, Kamonpan Wanichsri, Penpimol Ponchunchoovong, Suthasinee Thapphasaraphong, Natsajee Nualkaew

**Affiliations:** 1 Faculty of Pharmaceutical Sciences, Khon Kaen University, Khon Kaen 40002, Thailand; sineenad.te@kkumail.com (S.T.); kamonpan.w@kkumail.com (K.W.); penpimol.p@kkumail.com (P.P.); sutpit1@kku.ac.th (S.T.); 2 Faculty of Sciences, Khon Kaen University, Khon Kaen 40002, Thailand; alisa_n@kkumail.com

**Keywords:** mung bean, diabetic wound healing, intracellular ROS, cell proliferation, diabetic complication, zero waste

## Abstract

Mung bean seed coat (MBSC) is a byproduct of commercial vermicelli production that possesses essential activities capable of delaying diabetic wound progression. This study aimed to evaluate the potential of MBSC extracts to promote diabetic wound healing, an effect that has not yet been reported. The hypoglycemic effect was assessed by glucose uptake stimulation in L6 myotubes and by an α-glucosidase inhibition assay. Antiglycation was determined by BSA-glucose and BSA-methylglyoxal assays. Intracellular reactive oxygen species (ROS) reduction and wound healing were assessed in human keratinocytes (HaCaT) exposed to high glucose (HG), and gene expression in HG-wounded cells was analyzed by qPCR. The results demonstrated that the ethanolic extract (EE) from MBSC exhibited glucose-lowering effects and suppressed glycation reactions at the early and intermediate stages, with IC_50_ values of 75 and 140 µg/mL, respectively. EE reduced ROS by 70%, stimulated cell proliferation by 57% in the high-glucose (HG)-exposed HaCaT cells, and accelerated cell migration to close the HG-exposed wound. EE increased the gene expression of Nrf2, NQO-1, SOD2, and CAT. It also downregulated TNF-α, upregulated TGF-β1, and downregulated MMP-9. In conclusion, EE has the potential to delay the progression of diabetic wounds by lowering blood glucose levels, inhibiting AGE and ROS formation, and enhancing cell proliferation and migration in HG-exposed HaCaT cells. The gene regulatory effects of EE were demonstrated as an Nrf2 activator that reduced oxidative stress, exerted anti-inflammatory effects, and regulated ECM balance. The preparation of oral and topical products could be further developed.

## 1. Introduction

Type 2 diabetes mellitus (T2DM) is associated with chronic complications, such as delayed wound healing, diabetic retinopathy, nephropathy, peripheral arterial vascular disease, and diabetic neuropathy [1]. Among these, diabetic wounds are serious and increase the risks of mortality and disability. The impairment of wound healing results from chronic hyperglycemia, which promotes excess reactive oxygen species (ROS) production, the accumulation of advanced glycation end products (AGEs), chronic infection, and impaired angiogenesis [2]. ROS and AGEs cause oxidative stress and chronic inflammation, impairing the proliferation and migration of keratinocytes to close the wound [2]. Therefore, the wound-healing process is obstructed at the inflammation stage, during which elevated secretion of pro-inflammatory cytokines, such as interleukin-1 (IL-1), IL-6, IL-8, and tumor necrosis factor-α (TNF-α), occurs, and fails to progress to the re-epithelialization and remodeling stages [3]. Oxidative stress induced by hyperglycemia also elevates matrix metalloprotease (MMP) levels, particularly MMP-9, which degrades extracellular matrix (ECM) components, while reducing growth factors that promote ECM synthesis, such as transforming growth factor-β1 (TGF-β1) [4]. This imbalance between ECM formation and degradation critically disrupts re-epithelialization and further impairs the healing process of diabetic wounds [5].

Glycation, also known as the Maillard reaction, is a non-enzymatic reaction that involves the interaction between the carbonyl group of reducing sugars and the amine group of proteins. This process leads to the formation of dicarbonyl intermediates, such as methylglyoxal (MGO), which then react with amino acid residues in proteins, generating irreversible AGEs [6]. The accumulation of AGEs promotes oxidative stress, chronic inflammation, retards wound healing [6], and disrupts matrix remodeling [7]. Therefore, AGEs have become therapeutic targets for mitigating complications associated with diabetes mellitus (DM), as seen in the role of milk thistle seed extract [8]. Herbs that prevent glycation to AGEs are gaining interest for the prevention of diabetic complications, e.g., *Capsicum frutescens* and *Curcuma longa* [9]. The known AGE inhibitor, D-mannose, can maintain normal keratinocyte functions [10].

ROS, such as superoxide anion radicals, hydroxyl radicals, hydrogen peroxide, and perchloric acid, contribute to oxidative stress. In normal wounds, the nuclear factor erythroid 2-related factor 2 (Nrf2) pathway is a crucial mechanism for countering oxidative stress. Under normal conditions, Nrf2 binds to Kelch-like ECH-associated protein 1 (Keap1), and when the antioxidant response to ROS elimination is required, Nrf2 dissociates from Keap1 and translocates to the nucleus. There, it binds to and promotes the gene expression of antioxidant response elements (AREs), such as heme oxygenase-1 (HO-1), NAD(P)H) quinone oxidoreductase-1 (NQO-1), superoxide dismutase (SOD), and catalase (CAT) [11,12]. Nrf2 signaling pathway also contributes to anti-inflammatory effects. The binding of free Nrf2 to HO-1 in the nucleus leads to the inhibition of the NF-κB pathway and results in decreased expression of pro-inflammatory cytokines [13].

In diabetic wounds, chronic ROS elevation suppresses Nrf2 activity, impairing keratinocyte proliferation and migration and delaying wound closure. Restoration of Nrf2 activity induces antioxidant gene expression and enhances keratinocyte proliferation and migration, leading to accelerated wound healing [14]. Thus, an effective bioactive material for diabetic wound healing should have multifunctional properties. Among the key regulatory mechanisms, Nrf2 is highly expressed in skin keratinocytes and serves as a master regulator of the antioxidant response.

An Nrf2 activator accelerates diabetic wound healing by reducing oxidative stress [11]. It functions by binding to Nrf2, stabilizing it and inhibiting its degradation, or by regulating upstream signaling pathways that induce Nrf2 translocation into the nucleus. It also blocks the protein–protein interaction between Nrf2 and Keap1, maintaining or improving human keratinocyte cell viability and proliferation, increasing cell migration, and enhancing in vitro wound closure [11]. The diabetic wound is under greater oxidative stress than normal wound skin tissue, and Nrf2 activation is higher in the perilesional skin of diabetic patients than in normal skin.

HO-1 is also a target for reducing damage in diabetic complications. Inducing HO-1 expression and activity can enhance its antioxidant, anti-inflammatory, and anti-apoptotic effects, thereby reducing the extent of damage in diabetic complications [15]. Topical application of *Gynura divaricate* extract accelerates diabetic wound healing by activating the Nrf2 signaling pathway and upregulating HO-1 in diabetic rats [16].

Therefore, the targets of diabetic wound treatment should include enhancing cell proliferation and migration, anti-inflammation, antioxidant activity, reduction of oxidative stress by lowering ROS, and antiglycation. Natural sources associated to those targets include *Centella cordifolia* extract, which possesses strong antioxidant and antiglycation activities [17], berberine, which exerts anti-inflammation and antioxidant effects, and reduces oxidative stress [18] and D-mannose, a potent anti-inflammatory monosaccharide that has been developed as a topical product for the diabetic wound treatment in mice and accelerates wound healing by inhibiting AGEs production and upregulating AMPK/Nrf2/HO-1 in the high glucose environment [10].

Mung bean seed coat (MBSC) is obtained from mung bean (*Vigna radiata*). It is a waste product from vermicelli production. Many studies have been conducted on MBSC for functional food and health product development due to its major constituents, vitexin (apigenin-8-C-glucoside) and isovitexin (apigenin-6-C-glucoside). Both compounds have diverse biological activities, including potent antioxidant and anti-inflammatory effects [19], indicating multitarget effects to mitigate diabetes and its complications [20,21]. Isovitexin is a potential substance for diabetic wound healing by significantly promoting angiogenesis and vascular maturation, reducing oxidative damage, inhibiting apoptosis, and promoting collagen maturation [22].

Despite these promising biological profiles, no study has investigated MBSC extracts in the context of diabetic wound healing. MBSC, a byproduct from the food industry, contains abundant vitexin and isovitexin, bioactive compounds with pharmacological activities relevant to these processes. Therefore, this study aimed to evaluate the potential of MBSC extracts for diabetic wound healing by assessing hypoglycemic activity in L6 myotubes and wound-healing mechanisms in a high-glucose-exposed HaCaT keratinocyte model, as a basis for further development of oral and topical applications targeting the reduction of risk and delayed progression of early-stage diabetic ulcers.

## 2. Materials and Methods

### 2.1. Chemicals and Reagents

All general solvents were of analytical grade. Folin–Ciocalteu’s reagent, NaNO_3_, AlCl_3_, quercetin (purity ≥ 95%), ninhydrin reagent (cat. No. N7285), glycine (purity ≥ 99%), resorcinol, D-mannose (purity ≥ 95.0%), vitexin (purity ≥ 95.0%), isovitexin (purity ≥ 98%), 2,2′-azino-bis-(3-ethylbenzo-thiazoline-6-sulfonic acid) (ABTS), Trolox (purity ≥ 98%), 2,4,6-tris(2-pyridyl)-s-triazine (TPTZ) reagent, α-glucosidase, *p*-nitrophenyl-α-D-glucopyranoside (PNPG), bovine serum albumin (BSA), methylglyoxal (MGO), glucose, aminoguanidine HCl (AMG), sodium azide, 2′,7′-dichlorodihydrofluorescein diacetate (DCFH-DA), and fetal bovine serum (FBS) were purchased from Sigma–Aldrich (St. Louis, MO, USA). Gallic acid (purity > 98.0%) was purchased from Merck (Damstadt, Germany). Horse serum (HS) and antibiotic–antimycotic were purchased from Gibco (Thermo Fisher Scientific, Grand Island, NY, USA). α-Minimum essential medium (α-MEM) and 3-(4,5-dimethylthiazol-2-yl)-2,5-diphenyl-2H-tetrazolium bromide (MTT) were purchased from Invitrogen (Carlsbad, CA, USA). Dulbecco’s Modified Eagle Medium (DMEM) was purchased from Hyclone (Logan, UT, USA). The CO_2_ used in this study had a purity of 98%.

### 2.2. Mung Bean Seed Coat and Extraction

Mung bean (*Vigna radiata* L.) seed coats were purchased from a vermicelli factory in Ratchaburi, Thailand. They were dried in a hot air oven at 45 °C for 24 h, ground to a fine powder, and sieved through an 80-mesh sieve. Dried mung bean seed coat powder (80 g) was extracted with 80% EtOH (800 mL) in a shaker incubator at 50 °C, 90 rpm, for 12 h. The extract was filtered through a Buchner funnel using filter paper (Whatman No. 1, Cytiva, Bucks, UK), concentrated using a rotary evaporator, and subsequently freeze-dried to obtain the ethanolic extract (EE).

The seed coat powder (80 g) was boiled in deionized water (DI water) (800 mL) at 100 °C for 30 min. The filtrate was freeze-dried to obtain the aqueous extract (AE). To precipitate the mung bean polysaccharide, 6.19 g of AE was dissolved in 16 mL of DI water, followed by the addition of 32 mL of EtOH to a final concentration of 80% EtOH (*v*/*v*), and the mixture was kept at 4 °C for 24 h. The supernatant was collected and dried to yield the low-molecular-weight part (LMW). The precipitate was further redissolved in DI water and dialyzed against DI water using a regenerated cellulose tubular membrane (MWCO: 3500 Da; Cellu-Sep T1, Membrane Filtration Products, Seguin, TX, USA) for 48 h. The retentate was collected and freeze-dried to obtain the crude polysaccharide (CP).

### 2.3. Chemical Characterization of Mungbean Extracts

#### 2.3.1. Determination of Total Phenolic Content by Folin–Ciocalteu Assay

The reaction was performed in a 96-well plate containing 20 µL of the sample or gallic acid solutions, 100 µL of 10% (*v*/*v*) Folin–Ciocalteu reagent, and 80 µL of 7% (*w*/*v*) Na_2_CO_3_. The mixture was gently mixed and incubated in the dark at room temperature for 30 min. Absorbance was measured at 760 nm using a microplate reader (EnSight Multimode Plate Reader, PerkinElmer, Waltham, MA, USA). The total phenolic content (TPC) was calculated from the gallic acid standard curve over a concentration range of 15.625–250 µg/mL (y = 0.0056x + 0.0024, R^2^ = 0.9986), and was expressed as mg gallic acid equivalent per gram of extract (mg GAE/g extract).

#### 2.3.2. Determination of Total Flavonoid Content by Aluminum Chloride Colorimetric Method

The reaction was performed in a 96-well plate containing 100 µL of the sample, 20 µL of 5% (*w*/*v*) NaNO_3_, and 35 µL of 10% (*w*/*v*) AlCl_3_. The mixture was incubated in the dark at room temperature for 30 min, and absorbance was measured at 430 nm using a microplate reader (EnSight Multimode Plate Reader, PerkinElmer, Waltham, MA, USA). The total flavonoid content (TFC) was calculated from the quercetin standard curve over a concentration range of 15.625 to 250 µg/mL (y = 0.0046x + 0.0173, R^2^ = 0.998), and expressed as mg quercetin equivalent per gram of extract (mg QE/g extract).

#### 2.3.3. Determination of Total Amino Acid Content by Ninhydrin Assay

The ninhydrin assay was performed according to the manufacturer’s protocol, with modifications for the reaction in a 96-well plate. The reaction consisted of a sample or standard solution (75 µL) and ninhydrin reagent (37.5 µL). The mixture was mixed thoroughly and heated in a water bath at 100 °C for 10 min. After cooling, 187.5 µL of 95% EtOH was added. The absorbance was measured at 570 nm using a microplate reader (EnSight Multimode Plate Reader, PerkinElmer, Waltham, MA, USA). The total amino acid content was calculated from the glycine standard curve over a concentration range of 31.25–1000 µg/mL (y = 0.0014x + 0.0008, R^2^ = 0.9829) and was expressed as mg of glycine equivalent per gram of extract (mg GE/g extract).

#### 2.3.4. Determination of Total Polysaccharide Content by the Resorcinol–Sulfuric Acid Method

The polysaccharide content was determined using the resorcinol–sulfuric acid method as described by [23] with slight modifications. The reaction was performed in a 96-well plate with 25 µL of sample, 25 µL of resorcinol reagent, and 75 µL of concentrated H_2_SO_4_. The reaction mixture was gently mixed and incubated at 80 °C for 10 min. After cooling at room temperature, A_480_ was measured using a microplate reader (EnSight Multimode Plate Reader, PerkinElmer, Waltham, MA, USA). The total polysaccharide content was calculated from the D-mannose calibration graph (y = 0.0056x − 0.0064, R^2^ = 0.9974), and expressed as mg mannose equivalent per gram of extract (mg ME/g extract).

#### 2.3.5. High-Performance Liquid Chromatography (HPLC) Analysis

The HPLC analysis was performed using an HPLC instrument with an autosampler (Agilent 1260, Agilent Technologies, Santa Clara, CA, USA). The peaks were monitored using a UV diode array detector (DAD) at 360 nm. The HPLC column was an RP-18 column (Synergi™ 4 µm Fusion-RP 80 Å, LC column 250 × 4.6 mm, 5 µm, Phenomenex, Torrance, CA, USA). The sample volume was 10 µL, and the flow rate was set to 1 mL/min. The mobile phase consisted of solvent A: 0.2% (*v*/*v*) acetic acid in water and solvent B: 0.2% (*v*/*v*) acetic acid in acetonitrile, which were gradient-eluted as follows: at 0 min: 95% A; 25 min: 50% A; 35 min: 15% A; and at 45 min: 95%A, then the column was equilibrated for 5 min in 95% A. The relative content of vitexin and isovitexin (mg/g extract) was obtained from the standard graph (y = 30.094x − 30.318, R^2^ = 0.9991, and y = 19.758x − 13.167, R^2^ = 1.0000, respectively).

### 2.4. Antioxidant Assays

#### 2.4.1. ABTS Radical Scavenging Assay

The ABTS radical cation was generated by mixing 7 mM ABTS solution with 2.45 mM K_2_S_2_O_8_ (1:1) and incubating the mixture in the dark at room temperature for 12–16 h. Then, the ABTS^•+^ solution was diluted with MeOH to achieve A_734_ of 0.70 ± 0.02. The assay was performed in a 96-well plate containing 50 µL of sample and 150 µL of ABTS^•+^ solution. The reaction mixtures were incubated in the dark at room temperature for 30 min and measured at 734 nm using a microplate reader (EnSight Multimode Plate Reader, PerkinElmer, Waltham, MA, USA). The percentage of radical scavenging activity was calculated, and the IC_50_ was presented and compared with that of Trolox.

#### 2.4.2. Ferric Reducing Antioxidant Power (FRAP) Assay

The freshly prepared FRAP reagent, containing 300 mM sodium acetate buffer (pH 3.6), 10 mM TPTZ solution, and 20 mM FeCl_3_, in a 10:1:1 (*v*/*v*/*v*) ratio, was used. The reactions in a 96-well plate consisted of 50 µL of sample and 150 µL of FRAP reagent. They were incubated in the dark for 15 min and measured at A_593_ using a microplate reader (EnSight Multimode Plate Reader, PerkinElmer, Waltham, MA, USA). The antioxidant capacity was calculated from the Trolox standard calibration curve (1.625–250 µg/mL) and expressed as mg of Trolox equivalents per g of extract (mg TE/g extract).

### 2.5. α-Glucosidase Inhibition Assay

The α-glucosidase inhibitory activity was evaluated in a 96-well microplate according to the method of [24] with minor modifications. The reaction containing 50 μL of acarbose or extract, 15 μL of 0.1 U/mL α-glucosidase solution, and 20 μL of sodium phosphate buffer (pH 6.8) was initially incubated at 37 °C for 10 min; after that, 15 μL of 0.5 mM PNPG was added and incubated at 37 °C for 12 min. The reaction was terminated by adding 150 μL of 1 M Na_2_CO_3_, and the absorbance was measured at 405 nm using a microplate reader (EnSight Multimode Plate Reader, PerkinElmer, Waltham, MA, USA). The percentage of α-glucosidase inhibition was calculated using the following equation:

% α-Glucosidase inhibition = ((A_control_ − A_sample_)/A_control_) × 100

where A_control_ is the absorbance of the reaction without the test sample, and A_sample_ is the absorbance in the presence of the extract. The results were reported as IC_50_ values and compared with those of the positive control, acarbose.

### 2.6. Antiglycation Assays

#### 2.6.1. BSA-Glucose Assay

The BSA-glucose assay method was slightly modified from that described by [25]. The reaction in a 5 mL microcentrifuge tube consisted of 1 mL of 10 mg/mL BSA, 1 mL of 500 mM glucose, and 1 mL of the sample, in 0.2 M sodium phosphate buffer (pH 7.4). It was incubated at room temperature for 5 min, then 0.5 mM sodium azide (0.5 mL) was added, and it was further incubated in the dark at 37 °C for 7 days. The fluorescence intensity of the AGE product was measured at 370/440 nm using a microplate reader (Omega, BMG Labtech, Ortenberg, Germany). Aminoguanidine (AMG), an inhibitor of AGE formation, was used as a positive control. The percentage of glycation inhibition was calculated as follows:

% BSA-glucose glycation inhibition = [(F_control_ − F_sample_)/F_control_] × 100

where F_control_ is the fluorescence intensity of the reaction without a sample, and F_sample_ is the fluorescence intensity of the reaction with a sample.

#### 2.6.2. BSA-MGO Assay

The BSA-MGO method was performed in a 5 mL microcentrifuge tube using the method described by [25]. The reaction mixture (3 mL) consisted of 20 mg/mL BSA (1 mL), 60 mM MGO (1 mL), and the sample or standard (1 mL) in 0.1 M sodium phosphate buffer, pH 7.4. AMG was used as a positive control. The mixture was incubated at room temperature for 5 min, then 0.02% sodium azide (0.5 mL) was added, and incubated at 37 °C in the dark. An aliquot of 100 µL was applied to a black 96-well plate and measured for the fluorescence intensity at 370/440 nm using a microplate reader (EnSight Multimode Plate Reader, PerkinElmer, Waltham, MA, USA), on days 7, 14, and 21. The percentage of inhibition of glycation on the same day was calculated as follows:

% BSA-MGO glycation inhibition = [(F_control_ − F_sample_)/F_control_] × 100,

where F_control_ is the fluorescent intensity of the reaction containing BSA and MGO, and F_sample_ is the fluorescence intensity of the reaction containing BSA, MGO, and the sample.

### 2.7. Cell Culture Assays

#### 2.7.1. MTT Assay in L6 Myotubes

L6 myoblast cells (CRL-1458) were purchased from ATCC (Manassas, VA, USA). Cells were cultured in α-minimum essential medium (α-MEM) supplemented with 10% FBS and 1% antibiotic–antimycotic at 37 °C with 5% CO_2_. Cells (1.5 × 10^4^ cells per well) were seeded into 24-well plates and were differentiated into myotubes by changing the serum from 10% FBS to 2% HS.

The non-cytotoxic concentration range of the extracts was determined using the MTT assay. L6 myotubes were treated with various concentrations of the extracts in 24-well plates and incubated at 37 °C with 5% CO_2_ for 24 h. After treatment, the culture medium was removed, and cells were incubated with 0.5 mg/mL MTT solution for 2 h. DMSO was added to dissolve formazan crystals, and the absorbance at 570 nm was measured. Cell viability was calculated as a percentage. The concentration corresponding to 80% cell viability was considered the non-cytotoxic dose and used for subsequent experiments.

#### 2.7.2. Glucose Uptake Assay in L6 Myotubes

The extracts were dissolved in α-MEM supplemented with 2% HS and 1% antibiotic–antimycotic solution. L6 myotubes were treated with mung bean seed coat extracts at various concentrations for 48 h, and 200 nM insulin was used as a positive control. Medium glucose was quantified using a commercial glucose assay kit (GAGO kit, Sigma–Aldrich, St. Louis, MO, USA) according to the manufacturer’s protocol. The glucose concentration was calculated from the glucose standard calibration graph (y = 0.0039x + 0.0473, R^2^ = 0.999). Glucose uptake was determined from the reduced glucose concentration in the medium relative to the basal (untreated control) after 48 h of incubation and expressed as the percentage of glucose uptake stimulation.

#### 2.7.3. Intracellular ROS and Cell Proliferation Assay in the High Glucose (HG)-Exposed HaCaT Cells

Human keratinocytes (HaCaT) were purchased from Cell Line Services (CLS, Eppelheim, Germany). They were cultured in complete DMEM, consisting of DMEM supplemented with 1% antibiotic–antimycotic solution and 10% FBS, and incubated at 37 °C with 5% CO_2_. DMEM containing 25 mM glucose was used as the normal glucose (NG) condition. The cells (2 × 10^4^ cells/well) were seeded in a 96-well black plate with a clear bottom (Thermo Fisher Scientific, Waltham, MA, USA) for 24 h, then incubated with samples in DMEM containing 40 mM glucose and without FBS. DMEM medium without additional glucose (normal glucose) was used as the untreated control. Vitexin, isovitexin, and berberine were used as positive controls. Cell viability was determined using Presto Blue cell viability reagent (Thermo Fisher Scientific, Waltham, MA, USA), and fluorescence intensity was measured at 560/590 nm. Then, cells were washed with PBS, and 100 µL of 20 µM DCFH-DA was added, and the cells were incubated in the dark at 37 °C for 45 min. The fluorescence intensity was measured at 485/535 nm and was normalized to the cell viability percentage. The results were reported as the percentage of intracellular ROS in the treatments compared to the control (untreated HG-exposed HaCaT cells).

#### 2.7.4. Scratch Assay in HG-Exposed HaCaT

The wound closure stimulation effect was determined by a scratch assay [26]. HaCaT cells (5 × 10^4^ cells/well) were seeded in a 6-well plate in complete DMEM with 10% FBS until 90% confluence. After that, the cells were incubated for 24 h to achieve 100% confluence in non-FBS DMEM. Cells were scratched with a 200 µL pipette tip in three replicate wells per treatment condition; cell debris was removed by washing with PBS; and cells were then incubated with samples containing 40 mM glucose in DMEM (HG medium) without FBS. After 24 h, cells were washed with PBS, and replaced with new HG-medium containing the sample to mimic daily topical treatment. Berberine 25 µM and 5% FBS were used as positive controls. Cells were photographed at 0, 12, 24, 36, and 48 h using a cell microscope (EVOS M5000 imaging system, Thermo Fisher Scientific, Waltham, MA, USA) with a 4× objective lens, in phase contrast mode, with the position fixed. The wound area (µm^2^) was analyzed using ImageJ (version 1.54g, National Institutes of Health, Bethesda, MD, USA), and the percentage of wound closure was further calculated as follows:

% Wound closure = ((A_b_ − A_a_)/A_b_) × 100

where A_b_ is the wound area at 0 h, and A_a_ is the wound area at any time (h).

#### 2.7.5. qPCR Analysis of the HG-Exposed HaCaT Wound

HaCaT cells were seeded in a 6-well plate at a density of 5 × 10^4^ cells/well. After reaching confluence, a wound was created by the method mentioned in the scratch assay. Then, cells were treated with samples in the serum-free, high-glucose medium (40 mM glucose) for 24 h. Berberine (25 µM) and 5% FBS were used as positive controls. Cells in high-glucose medium without FBS were used as the negative control (HG), and cells in DMEM without FBS were used as the untreated control (NG). Total RNA was extracted using TRIzol reagent (Thermo Fisher Scientific, Waltham, MA, USA) and quantified with a NanoDrop One spectrophotometer (Thermo Fisher Scientific, Waltham, MA, USA). cDNA was synthesized from 2 µg of total RNA using the RevertAid First Strand cDNA Synthesis Kit (Thermo Fisher Scientific, Vilnius, Lithuania). Quantitative real-time PCR was performed on a CFX96 Real-Time PCR Detection System (Bio-Rad, Singapore) using 2× Maxima SYBR Green qPCR Master Mix (Thermo Scientific, Vilnius, Lithuania). The primers for the targeted genes associated with antioxidant enzymes, inflammatory responses, and extracellular matrix (ECM) regulation, where β-actin was used as the housekeeping gene control, are listed in Table 1. Relative fold changes in gene expression were calculated using the 2^−ΔΔCT^ method [27].

### 2.8. Statistical Analysis

All experiments were performed in triplicate. Data were expressed as mean ± standard deviation (S.D.). Statistical differences between the treated groups and the control group were analyzed using one-way analysis of variance (ANOVA) (SPSS version 29, IBM, Armonk, NY, USA). A *p*-value < 0.05 was considered statistically significant.

## 3. Results

### 3.1. Extraction and HPLC Analysis

EE provided the highest yield. CP was the crude polysaccharide that precipitated from AE using 80% EtOH. The remaining supernatant contained low-molecular-weight compounds. Vitexin and isovitexin were dominant in EE and LMW, while they were present in trace amounts in CP. Although vitexin and isovitexin contents in EE and LMW were comparable (6.2% vs. 6.3%, and 14.4% vs. 16.7%, respectively), EE had a 1.9-fold higher yield relative to MBSC dry weight than LMW, as shown in Table 2.

**Figure 1:**
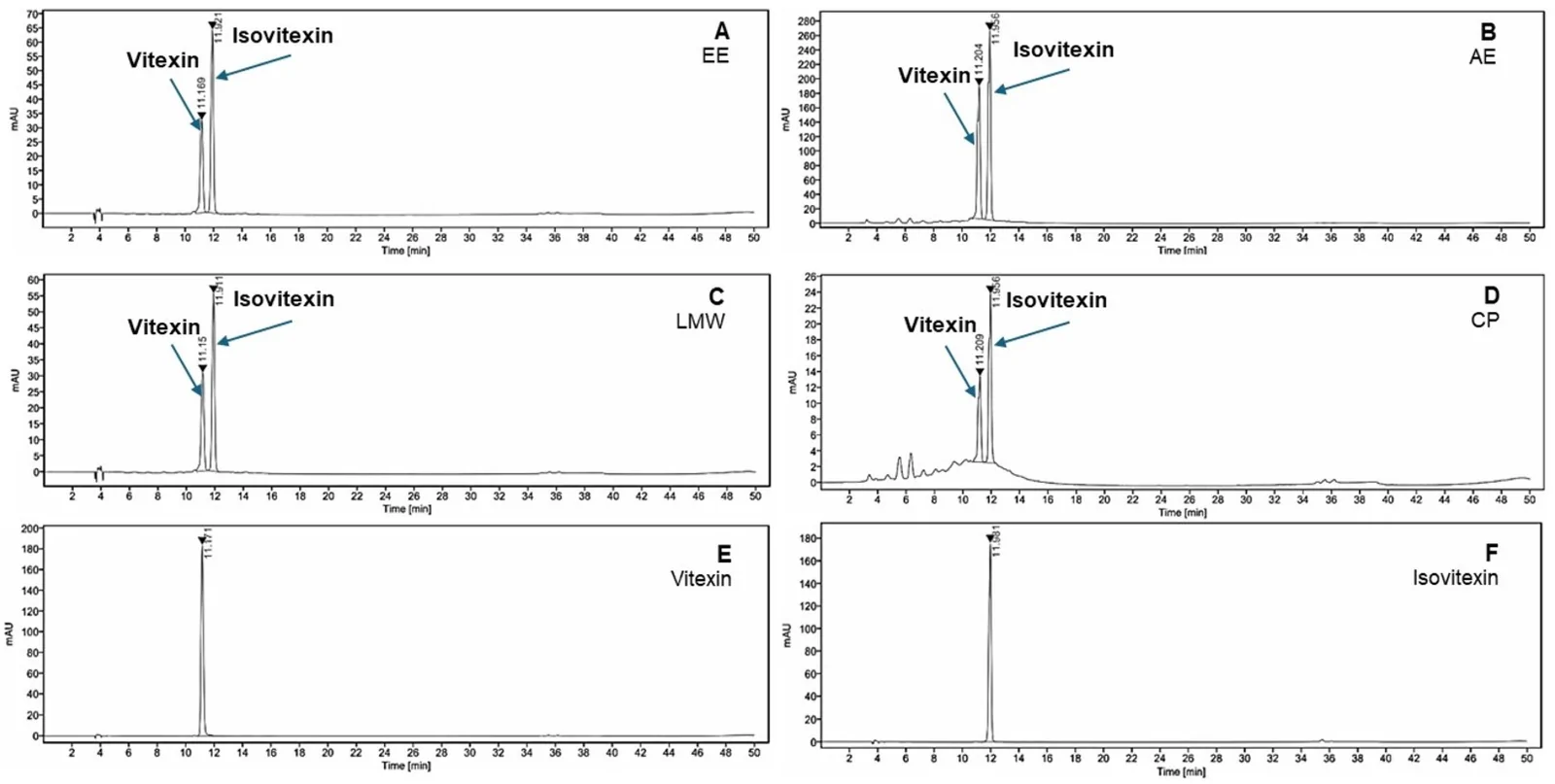
HPLC chromatogram of the mung bean extracts showing vitexin and isovitexin ((**A**): 0.25 mg/mL EE; (**B**): 10 mg/mL AE; (**C**): 0.25 mg/mL LMW; (**D**): 10 mg/mL CP; (**E**): 100 µg/mL vitexin; (**F**): 100 µg/mL isovitexin), absorbance at 360 nm, sample volume 10 µL.

The HPLC chromatogram of EE, AE, and LMW showed vitexin and isovitexin as the major components, with peaks at retention times of 11.171 and 11.981 min, respectively, while CP was present in trace amounts. AE and CP consisted of polar compounds, as shown by the peaks eluting before vitexin and isovitexin (Figure 1), which corresponded to the extraction process using water as the solvent.

### 3.2. Characterization of Mung Bean Seed Coat Extracts

The total phytochemical contents of the extracts are presented in Table 3. All extracts contained phenolic compounds, flavonoids, and amino acids. Polysaccharide levels were high in AE and CP. Since polysaccharides precipitate in EtOH, they were not determined in EE and LMW. A high TPC in CP suggested the presence of phenolic polysaccharide complexes.

### 3.3. Antioxidant and α-Glucosidase Inhibitory Effects of MBSC Extracts

All extracts exhibited moderate antioxidant and α-glucosidase inhibitory activities and were less potent than Trolox and acarbose, the positive controls, as shown in Table 4. LMW was the most potent antioxidant in the FRAP and ABTS assays. CP possessed the highest α-glucosidase inhibitory effect, although it contained the least amount of vitexin and isovitexin.

### 3.4. Antiglycation Properties of MBSC Extracts

#### 3.4.1. The Effect of MBSC Extracts on the BSA-Glucose Assay

**Figure 2:**
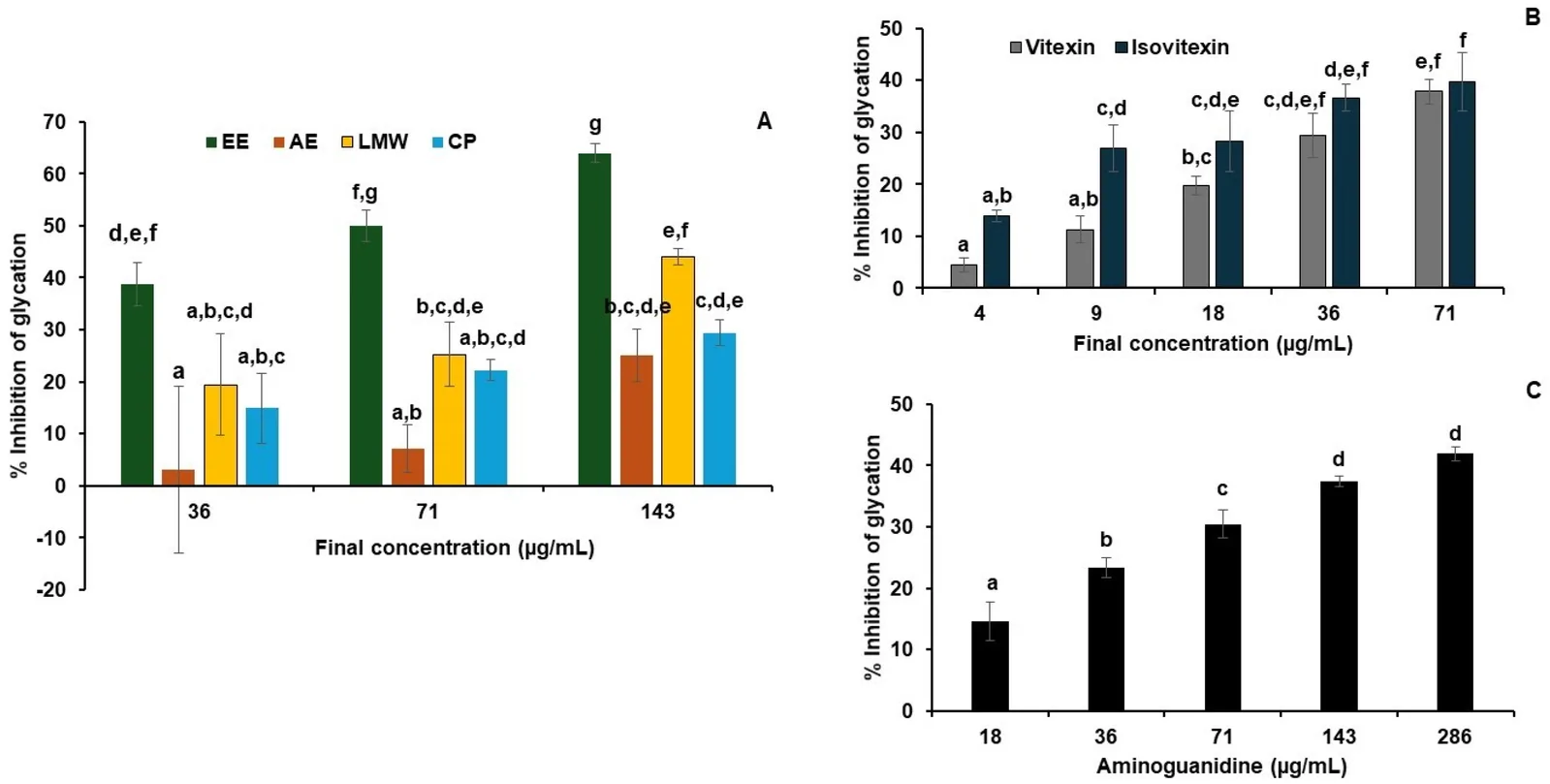
Antiglycation effect of MBSC extracts by the BSA-glucose assay ((**A**): MBSC extracts (EE, AE, LMW, CP); (**B**): vitexin and isovitexin (biomarkers); (**C**): aminoguanidine (positive control)). Data are presented as mean ± standard deviation (S.D.), different letters indicate significant differences between groups at *p* < 0.05 by Tukey’s multiple comparison test (*n* = 3).

The BSA-glucose assay demonstrated inhibition of glycation at an early stage. MBSC extracts, vitexin, isovitexin, and aminoguanidine exhibited antiglycation activity in the BSA-glucose assay in a dose-dependent manner (*p* < 0.05), as shown in Figure 2. Among MBSC extracts, EE was the most potent antiglycation extract, with an IC_50_ value of 75 µg/mL, compared with AE, LMW, and CP (Figure 2A).

#### 3.4.2. The Effect of MBSC Extracts on the BSA-MGO Assay

**Figure 3:**
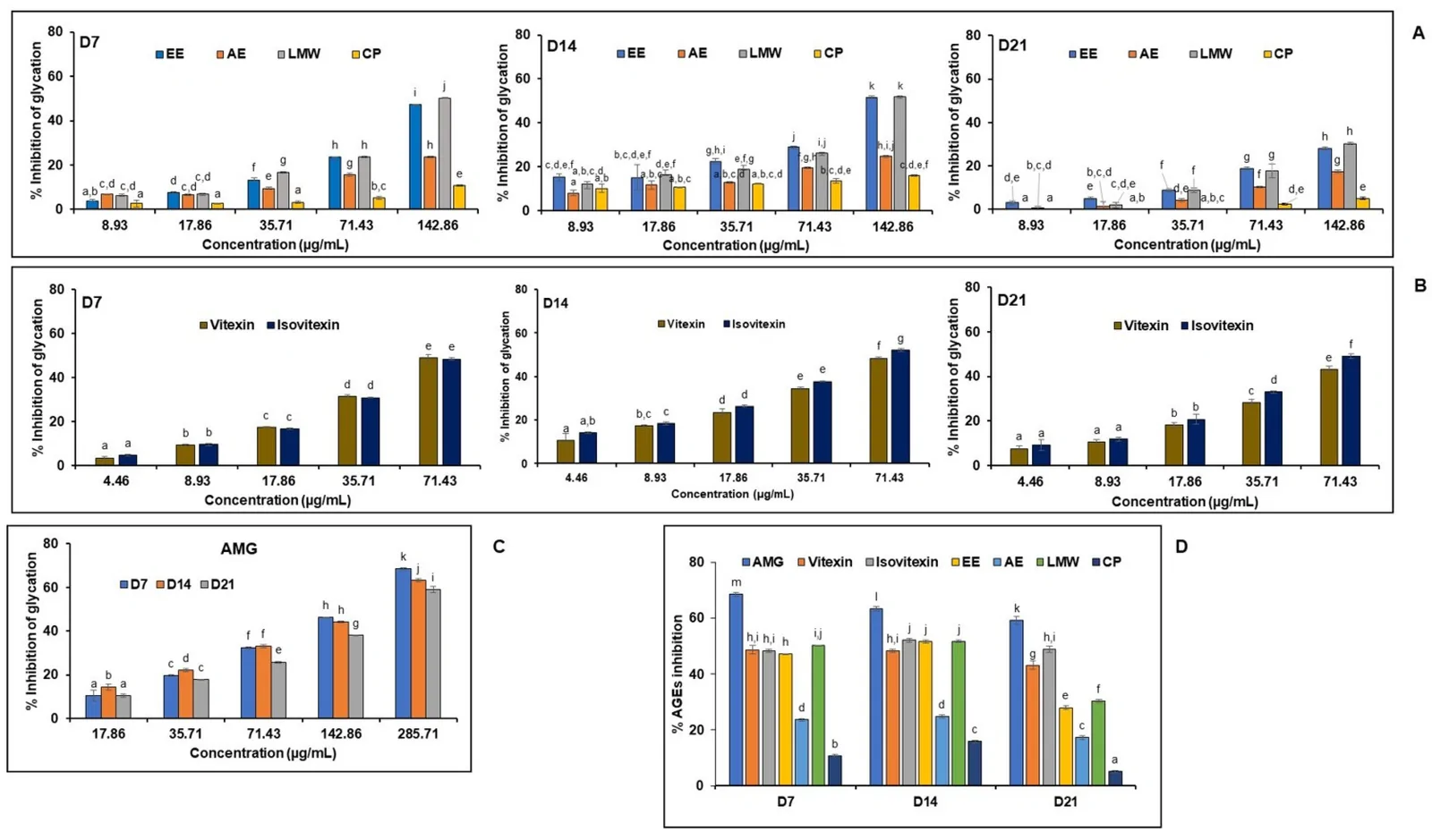
Glycation inhibitory effect of mung bean seed coat extracts at day 7, 14 and 21 by the BSA-MGO assay ((**A**): Dose-dependent effect of the MBSC extracts; (**B**): Dose-dependent effect of vitexin and isovitexin; (**C**): Dose-dependent effect of aminoguanidine; (**D**): Comparison of the glycation inhibitory effect among aminoguanidine, vitexin, isovitexin, and MBSC extracts at the highest effective concentrations 285.71, 71.43, 71.43, and 142.86 µg/mL, respectively, at day 7, 14, and 21). Data are presented as mean ± standard deviation (S.D.), different letters indicate significant differences between groups at *p* < 0.05 by Tukey’s multiple comparison test (*n* = 3).

The BSA-MGO assay demonstrated inhibition of glycation at the intermediate stage. The results showed that all samples exerted inhibitory effects in a dose-dependent manner (Figure 3), reaching a maximal level on day 14 and declining on day 21 (Figure 3A). AMG, the positive control, exhibited the highest inhibition, with IC_50_ values of 178.48, 193.37, and 225.32 µg/mL on days 7, 14, and 21, respectively. Vitexin and isovitexin, the active compounds of MBSC, were more potent than the MBSC extracts. The IC_50_ value of isovitexin on day 14 was 63.98 µg/mL, while those of EE and LMW were 140.04 and 142.37 µg/mL, respectively. CP showed only a mild antiglycation effect. The potency of AE to inhibit glycation was between those of LMW and CP. Comparison of the glycation inhibitory effects at the highest effective dose of AMG, vitexin, isovitexin, and MBSC extracts is demonstrated in Figure 3D, showing the potency of AMG > isovitexin > vitexin > LMW > EE > AE > CP. The results suggested that antiglycation ability was related to vitexin and isovitexin content.

### 3.5. The Glucose Uptake Effect of the MBSC Extracts in L6 Myotubes

**Figure 4:**
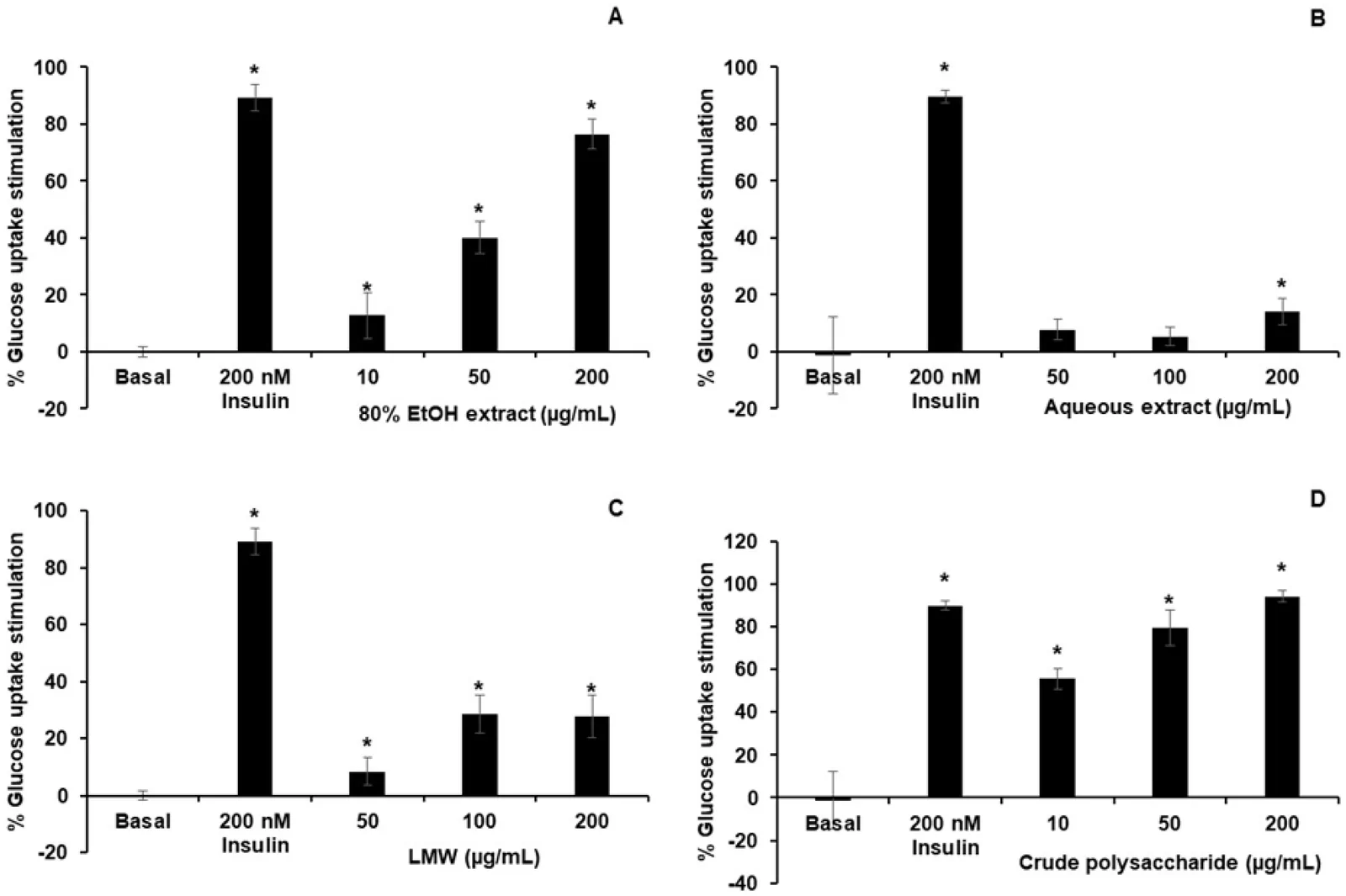
Glucose uptake stimulation effects of MBSC extracts in L6 myotubes at non-cytotoxic concentrations. (**A**): EE; (**B**): AE; (**C**): LMW; (**D**): CP. Data are presented as mean ± S.D.; * *p* < 0.05 compared with basal; *n* = 3.

At non-cytotoxic concentrations, all MBSC extracts significantly enhanced glucose uptake in L6 myotubes compared to the basal condition (untreated L6 myotubes). As glucose uptake normally occurs in untreated L6 myotubes, treatment that increases glucose uptake above basal levels indicates stimulatory properties. Insulin, 200 nM, showed 85% stimulation of glucose uptake. Vitexin and isovitexin, the abundant substances in MBSC, stimulated glucose uptake by 70–75% at 12.5 µg/mL, indicating their strong effect. CP exerted the most potent effects, despite the lowest levels of vitexin and isovitexin (Table 2), whereas LMW exhibited moderate activity, and AE showed only mild activity, as shown in Figure 4. At 200 μg/mL, EE, AE, LMW, and CP stimulated glucose uptake by 76, 14, 28, and 94%, respectively. The IC_50_ of EE was 81.14 µg/mL, while that of CP was less than 10 μg/mL. The results suggested that glucose uptake was not solely dependent on vitexin and isovitexin content, and that polysaccharides might also be active compounds.

### 3.6. Effects of the MBSC Extracts in HG-Exposed HaCaT Cells

#### 3.6.1. Effects of MBSC Extracts on NG-HaCaT Cell Proliferation

**Figure 5:**
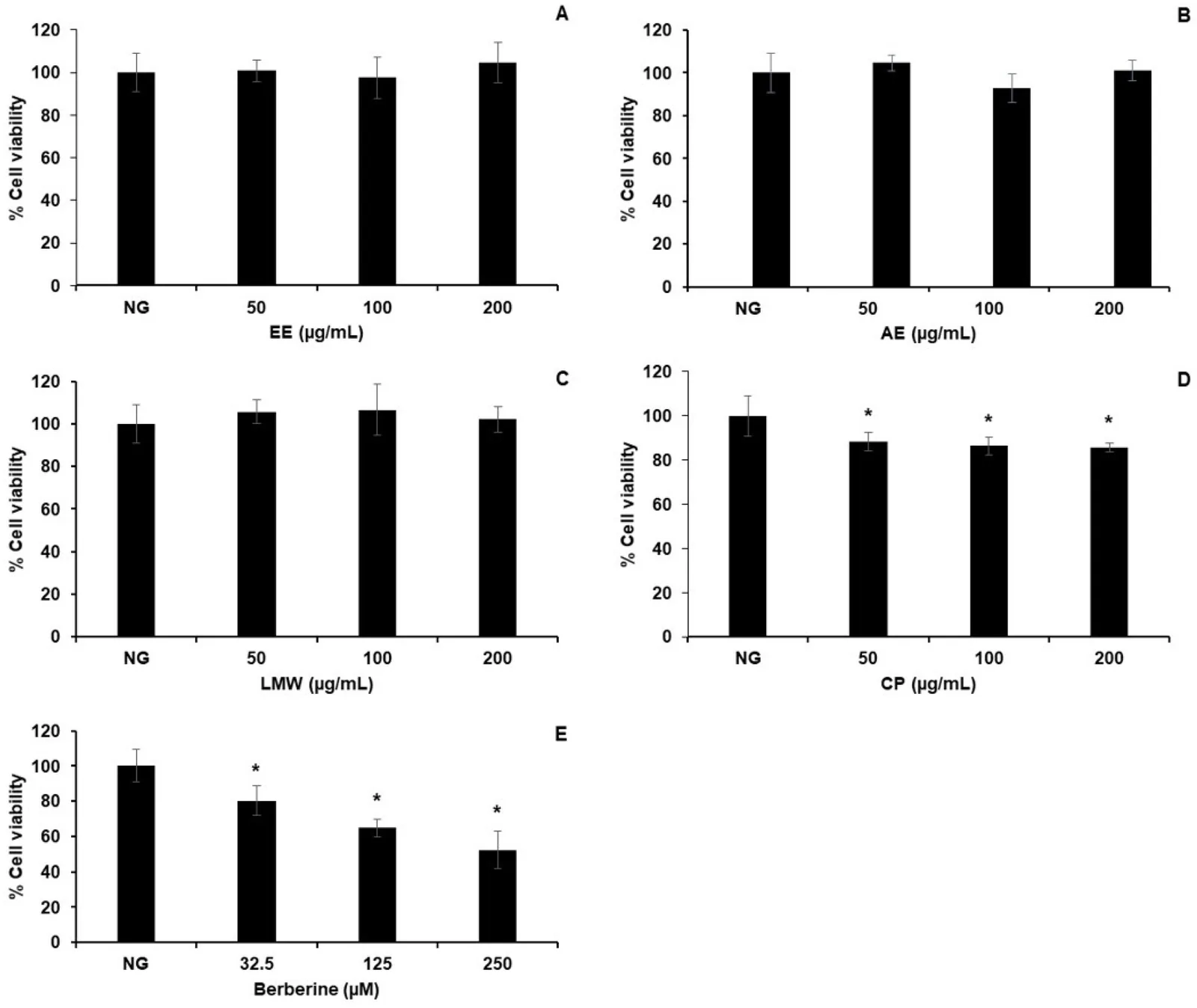
Effects of mung bean seed coat extracts on cell viability in HaCaT cells in the normal glucose medium (25 mM glucose). Cells were treated for 24 h and were evaluated by MTT assay, (**A**): EE; (**B**): AE; (**C**): LMW; (**D**): CP; (**E**): berberine (positive control). Data were presented as mean ± S.D.; * *p* < 0.05, comparing the untreated control (NG) and treatments, *n* = 4.

The MTT assay of MBSC extracts in normal glucose (NG)-treated HaCaT cells demonstrated cell viability of 80–100% at concentrations up to 200 µg/mL, indicating a non-cytotoxic concentration range without significant stimulation of cell proliferation (*p* < 0.05), as shown in Figure 5. Berberine, a ROS inhibitor, was used as a positive control and showed a non-cytotoxic concentration of 32.5 µM and no effect on cell proliferation.

#### 3.6.2. Mung Bean Seed Coat Extracts Attenuated the Intracellular ROS in the HG-Exposed HaCaT Cells

**Figure 6:**
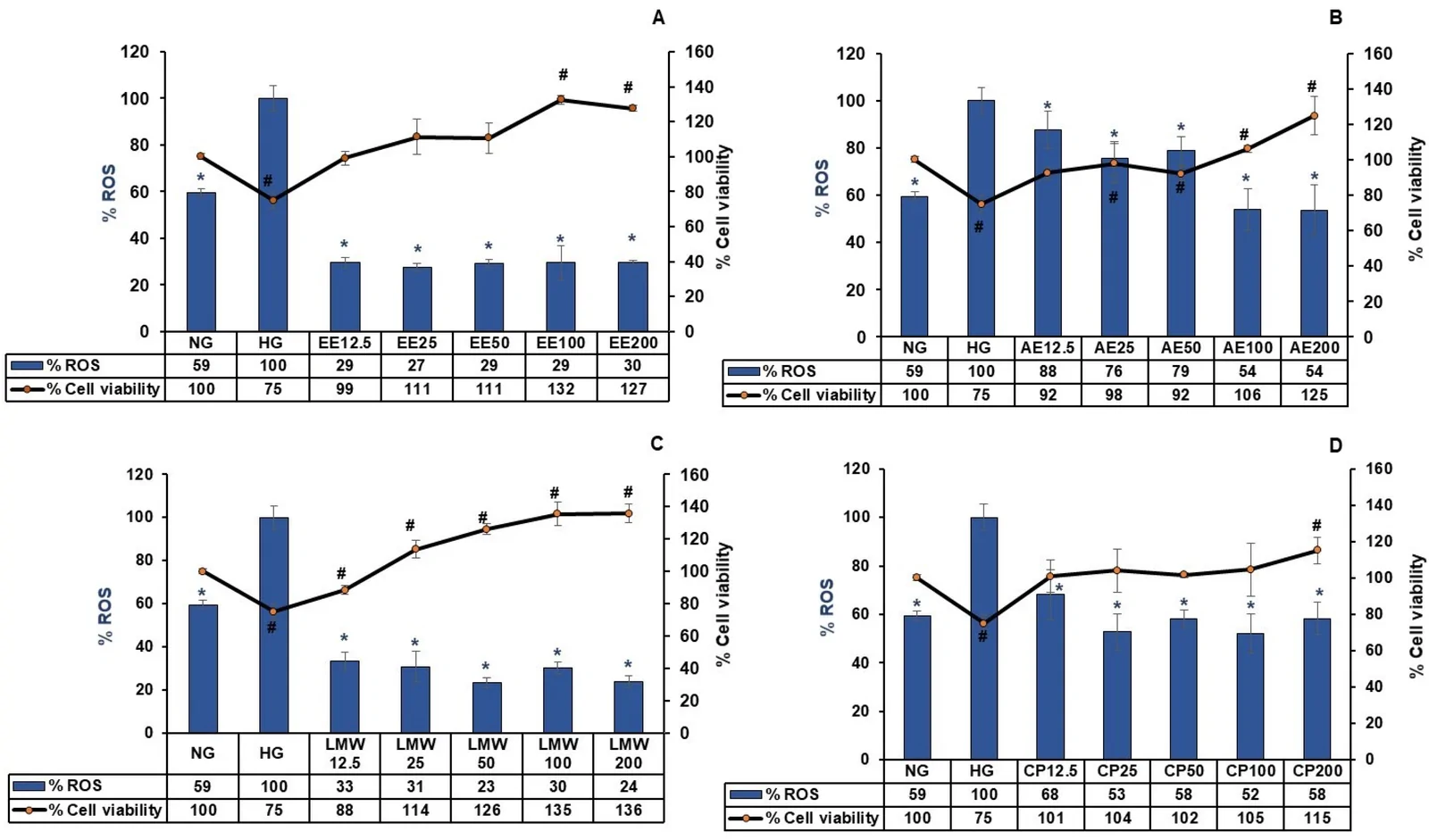
Effect of mung bean seed coat extracts at concentrations of 12.5–200 µg/mL to reduce intracellular ROS and enhance cell viability in HG-exposed HaCaT cells after 24 h treatment. Cell viability was measured using PrestoBlue. ((**A**): EE; (**B**): AE; (**C**): LMW; (**D**): CP). Data were presented as mean ± S.D.; * *p* < 0.05, comparing % ROS between the untreated HG-exposed HaCaT (% ROS of HG was set as 100%) and the normal condition (NG) and the treated HG-exposed HaCaT; ^#^ *p* < 0.05, comparing % cell viability between NG (100% cell viability) and the untreated HG-exposed HaCaT and treated HG-exposed HaCaT; *n* = 3.

Exposure of HaCaT cells to high glucose (40 mM glucose) for 24 h significantly increased ROS formation by 41% and reduced cell proliferation by 25% compared with the normal glucose (25 mM) condition (*p* < 0.05), as shown in Figure 6. MBSC extracts (EE, AE, LMW, and CP) not only restored cell viability but also significantly enhanced cell proliferation (*p* < 0.05) in HG-exposed cells by 50, 50, 61, and 40%. At this concentration, EE and LMW reduced intracellular ROS by 70% and 76%, respectively, which were greater reductions than those observed with AE and CP (46% and 42%, respectively). The results indicated the ability of the extracts to enhance cell viability under ROS-generating high-glucose conditions and to attenuate intracellular ROS, especially in extracts containing vitexin and isovitexin.

### 3.7. Wound Closure Effect of Mung Bean Seed Coat Extracts in HG-Exposed HaCaT Wound

**Figure 7:**
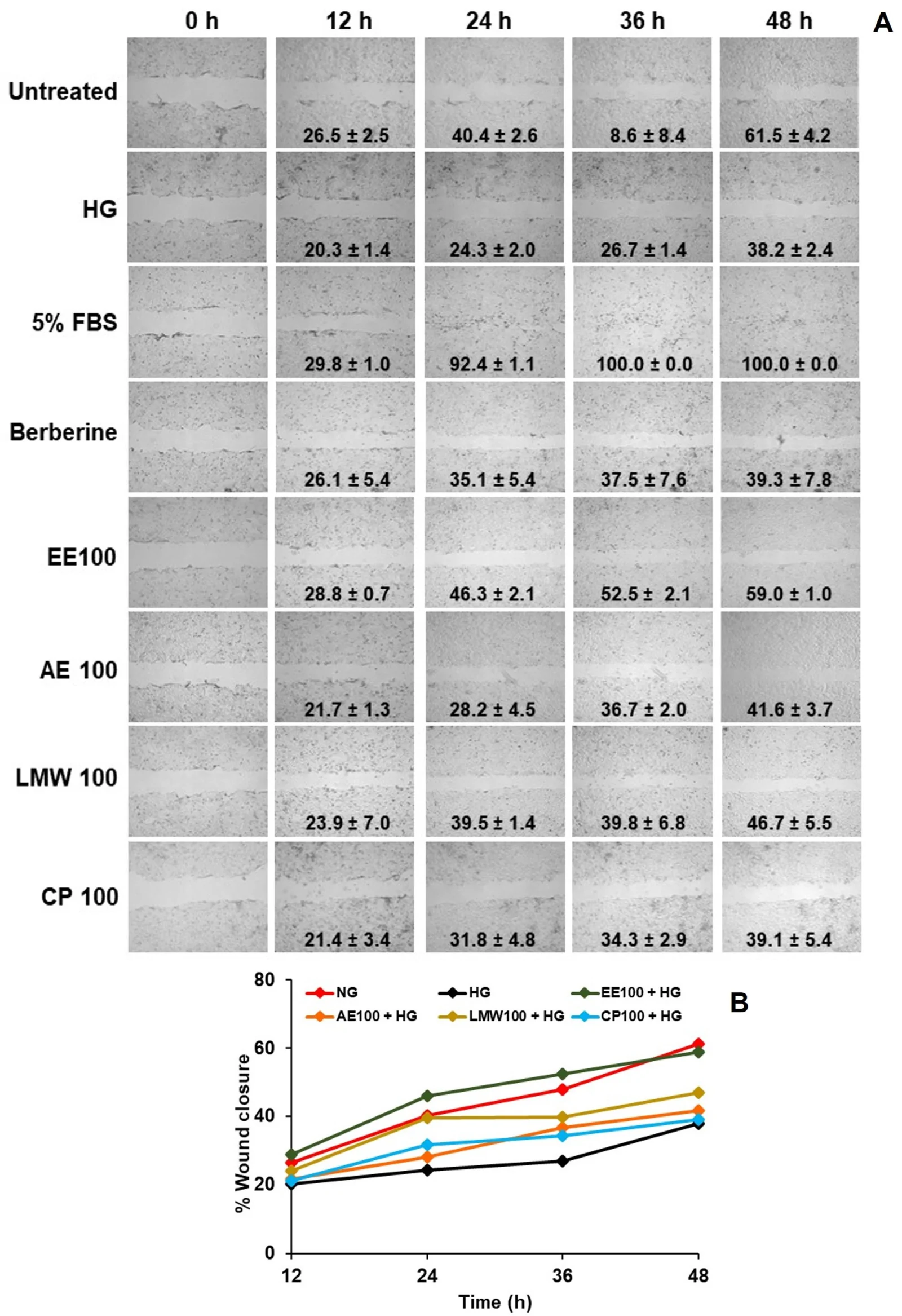
Wound closure effects of MBSC extracts in HG-exposed HaCaT cells. (**A**): HaCaT wounds were treated with MBSC extracts at a concentration of 100 µg/mL in the presence of 40 mM glucose for 0–48 h, compared with 5% FBS and 25 µM berberine in the presence of 40 mM glucose. The average percentage of wound closure ± S.D. was presented at 12, 24, 36, and 48 h. (**B**): Comparison of % wound closure of MBSC extracts at 100 µg/mL in HG-exposed HaCaT wounds at 12, 24, 36, and 48 h. Berberine (25 µM) and FBS (5%) were used as positive controls. The untreated control was HaCaT cells wounded under normal glucose conditions (HG: 40 mM glucose, NG: 25 mM glucose, *n* = 3).

HaCaT wounds in the presence of high glucose (HG, 40 mM glucose) showed 38% wound closure at 48 h after the scratch, indicating the effect of high glucose, which delays wound healing by suppressing cell migration, when compared to wounds in normal glucose (NG, 25 mM glucose) that showed 61.5% wound closure. FBS (5%) was used as a positive control and showed complete wound closure after 24 h of treatment, while berberine (25 µM) showed a percentage of wound closure similar to that in the presence of HG, indicating that it did not accelerate cell migration (Figure 7A). The wound area (µm^2^) and percentage of wound closure for MBSC extract treatments at concentrations of 50 and 100 µg/mL are shown in Table S1. Comparison of the percentage of wound closure among the mung bean seed coat extracts at 100 µg/mL (12–48 h) is shown in Figure 7B. EE demonstrated the most potential to promote wound closure in HG-exposed HaCaT cells by enhancing cell migration. LMW enhanced wound closure less effective than EE, while AE exhibited a mild effect at 24 and 36 h (Figure 7B). At 48 h treatments with MBSC extract at 100 µg/mL, EE provided 59% wound closure, which was not significantly different from the NG condition, indicating a recovery of cell migration toward normal level. The percent wound closure for AE and CP showed no significant difference compared to HG control, suggesting inactive effects at 48 h (Figure S1).

Since EE exhibited the best effects on antiglycation, enhancing cell proliferation, and reducing intracellular ROS in HG-exposed cells, it also highly promoted cell migration in the HG-exposed HaCaT wound; therefore, the effect of EE on gene expression related to diabetic wound healing functions, such as antioxidative stress, anti-inflammation, and regulation of ECM in the HG-exposed HaCaT wound compared to the wound under normal glucose conditions (NG), was further investigated.

### 3.8. Effects on mRNA Expression of Antioxidant Genes

**Figure 8:**
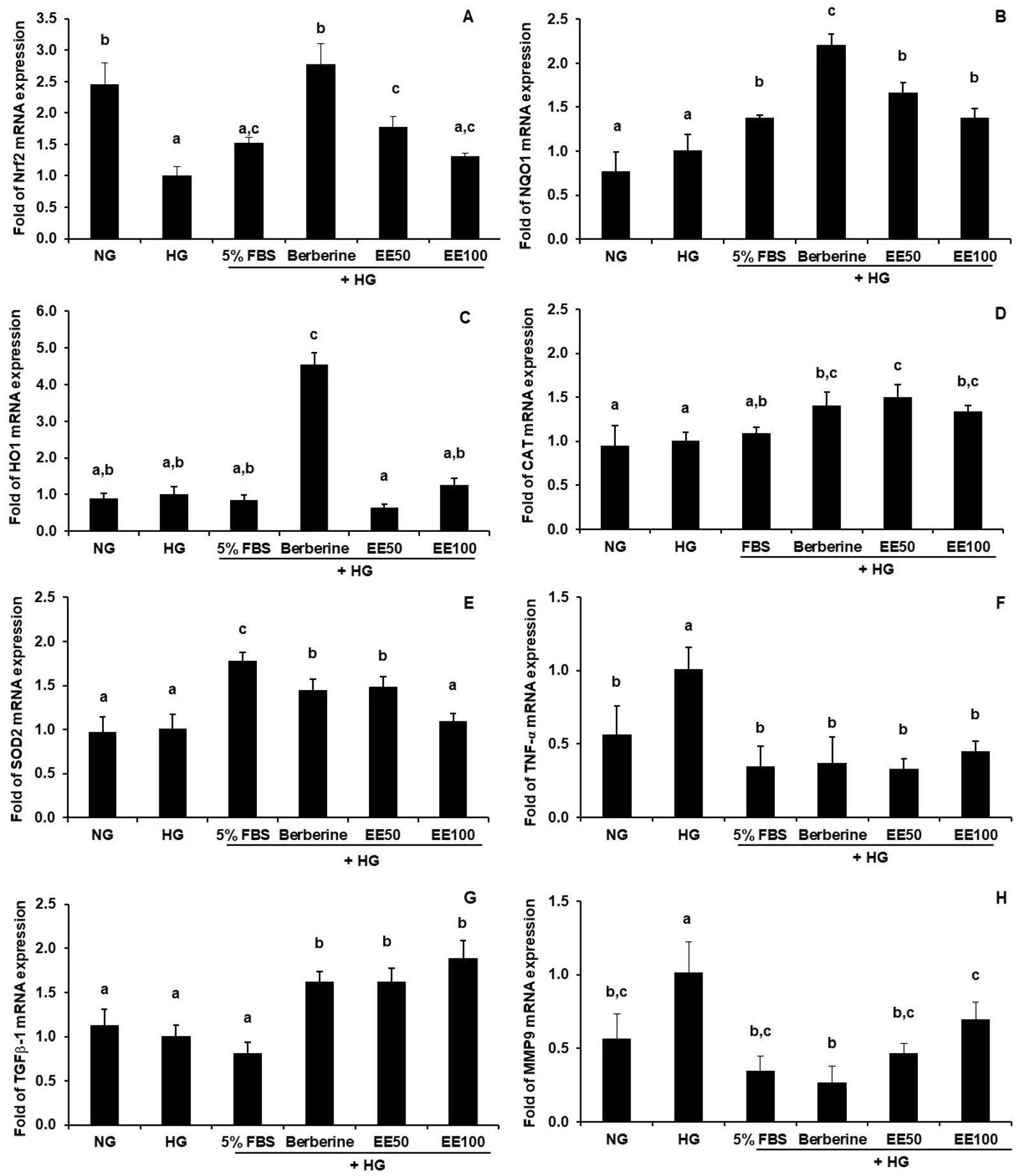
Effect of EE on the mRNA expression of genes in high-glucose-exposed HaCaT wounds after 24 h treatment. ((**A**): Nrf2; (**B**): NQO-1; (**C**): HO-1; (**D**): CAT; (**E**): SOD2; (**F**): TNF-α; (**G**): TGFβ-1; (**H**): MMP-9; NG: normal glucose; HG: high glucose (40 mM glucose); Berberine 25 µM was used as a positive control; EE: ethanolic extract; fold of mRNA expression was compared to the untreated HG-exposed HaCaT wound; different letters represented statistically different, *p* < 0.05, *n* = 3).

The HG-exposed wound showed 60% downregulation of the Nrf2 gene compared with the NG-exposed wound (*p* < 0.05). Treatment with 25 µM of berberine (positive control) and EE in the HG-exposed wound significantly enhanced the mRNA expression of Nrf2, NQO-1, CAT, and SOD2 compared to the untreated HG-exposed wound (*p* < 0.05), as shown in Figure 8A–E. The results indicated that EE functioned as an Nrf2 activator, leading to downstream expression of antioxidant enzymes in the Nrf2 pathway and contributing to reduced ROS levels (Figure 6A).

### 3.9. Effects on the Anti-Inflammatory and ECM Regulatory Gene Expression

The HG-exposed wound upregulated TNF-α and MMP-9 gene expression (*p* < 0.05) but did not alter TGF-β1 mRNA levels compared to the NG-exposed wound. Treatment with EE in the HG-exposed HaCaT wound significantly downregulated TNF-α (Figure 8F), indicating an anti-inflammatory effect. EE decreased MMP-9 gene expression (Figure 8H), suggesting the inhibition of ECM degradation by MMP-9. The upregulation of TGF-β1 by EE treatment (Figure 8G) was associated with its enhancement of cell migration to close the wound (Figure 7).

## 4. Discussion

Chronic high blood glucose leads to many subsequent complications, such as cataract, degenerative neurons, retinopathy, and chronic wounds. Diabetic wounds are one of the major complications of diabetes. Diabetic wounds differ from normal wounds by increased ROS production, which results in oxidative stress and prolonged inflammation, delaying re-epithelialization by suppressing cell proliferation, reducing cell migration, and elevating MMP levels. Keratinocytes are the first target cells in diabetic wound healing. Therefore, this study focused on diabetic wound healing in HaCaT cells exposed to high glucose.

Diabetic wounds progress from the pre-ulcer stage, with intact skin and inflammation, to a superficial ulcer without any impact on the tendon, capsule, or bone. Subsequently, they progress to a deep ulcer stage reaching the tendon, bone, or joint, and end up in whole-foot gangrene that requires amputation of the leg [32]. Substances that alleviate chronic hyperglycemia and reduce chronic inflammation and ROS have been considered to have high potential for inhibiting wound progression and, in turn, promoting cell proliferation and migration, attenuating inflammation, and reducing ECM degradation in diabetic keratinocytes.

Mung bean seed coat (MBSC) extracts consist mainly of vitexin and isovitexin, bioactive flavonoid glycosides with anti-diabetic, antioxidant, wound-healing, and anti-ROS activities [21], and also contain water-soluble polysaccharides with anti-inflammatory, antioxidant, and antibacterial activities [33,34]. Although diverse activities of MBSC extracts have been reported, no assessment of their potential to delay diabetic wound progression has yet been conducted.

In this study, four extracts from mung bean seed coats were prepared. Extraction with 80% EtOH was targeted at glycosides. Mung beans are rich in polysaccharides and phenolic compounds. The aqueous extract (AE) was designed to obtain polysaccharides and polar substances. Crude polysaccharide (CP) was obtained from the precipitation of AE and was then separated from the filtrate containing low-molecular-weight substances (LMW). HPLC chromatograms of the extracts showed peaks of vitexin and isovitexin as the dominant compounds in EE, AE, and LMW, with trace amounts in CP, which corresponded to the extraction processes.

The hypoglycemic activities of the MBSC extracts were demonstrated by glucose uptake-stimulatory and α-glucosidase-inhibitory effects, with varying potencies, indicating that multiple substances were responsible for these activities, not only vitexin and isovitexin. This was evident in LMW, which contained similar amounts of vitexin and isovitexin to EE but exhibited a weaker glucose uptake effect. CP, with trace amounts of vitexin and isovitexin, exerted the most potent glucose-lowering effects, including glucose uptake stimulation and α-glucosidase inhibition.

The lack of correlation between chemical content and the hypoglycemic potency of AE and CP was unexpected, which might be resulted by their bioactive constituents, and might result from the formation of polysaccharide (PS)–polyphenol (PP) complexes in those extracts, leading to an increase or decrease in the hypoglycemic activities of the extracts. There have been no previous reports of the effects of PS–PP complexes on the glucose uptake mechanism; rather, there are reports of the synergistic hypoglycemic effect of jujube PS–PP complexes, which enhance the α-glucosidase inhibitory activity compared with individual PS and PP [35], as well as reduced those activities by grape pomace PS–PP complexes [36].

Hyperglycemia leads to glycation that forms AGEs. AGEs delay diabetic wound healing by prolonging inflammation and increasing oxidative stress [37]. On day seven, EE exhibited a better antiglycation effect in the BSA-glucose model than in the BSA-MGO model, revealing that EE suppressed the initial stage of glycation that utilized glucose as a substrate better than the middle or late stages that utilized MGO as a substrate to form dicarbonyl intermediates and AGEs. In contrast, LMW inhibited the initial stage more slowly than the mid-stage. It was noted that EE and LMW, which are rich in vitexin and isovitexin, inhibited glycation more effectively than AE and CP, which are rich in polysaccharides and might have polysaccharide–phenolic complexes that weakened the antiglycation ability, as demonstrated by PS–PP conjugates from red grape pomace [36]. Vitexin and isovitexin are known potent antiglycation substances. Vitexin (500 μM) inhibits AGEs by more than 80% in both BSA-fructose and BSA-MGO models [38]. Isovitexin has a stronger antiglycation effect than aminoguanidine and inhibits glycation at both early and intermediate stages [39]. Aminoguanidine is an anti-AGE agent that inhibits glycation by 73.61% at 2 mg/mL in the BSA-glucose model [40]. Moreover, isovitexin could promote angiogenesis and vascular maturation, reduce oxidative damage and apoptosis, and improve collagen organization [22].

Wounds of keratinocytes in a high-glucose environment suppress cell proliferation, reduce cell migration to close the wound, elevate MMP levels, and increase ROS production [10,41]. In this study, wound-healing assays in human keratinocytes (HaCaT) were performed in a medium containing 40 mM glucose (HG) to mimic a hyperglycemic condition. The MBSC extracts (EE, AE, LMW, and CP) could restore cell viability, promote cell proliferation, and reduce ROS levels of HG-exposed HaCaT cells. These effects supported the wound-healing properties of EE and LMW in the HG-exposed HaCaT wound by enhancing cell migration. However, AE and CP did not promote cell migration, nor did berberine, a positive control for a diabetic wound-healing. This suggested different mechanisms of action for mitigating the factors underlying delayed diabetic wound healing. The strong effects of ROS reduction, cell proliferation, and cell migration in HG-exposed HaCaT were observed in EE and LMW, which had high vitexin and isovitexin contents, and the weaker effects of AE and CP might be due to lower vitexin and isovitexin contents.

ROS stimulate wound closure, cell migration, angiogenesis, and collagen production. However, in diabetic patients, ROS levels are elevated, leading to an imbalance between ROS production and elimination due to insufficient antioxidant enzymes (such as superoxide dismutase and catalase) [42]. MBSC aqueous extract possesses intracellular ROS reduction effects in insulin-resistant HepG2 cells, which involve the molecular mechanisms of increased expression of the antioxidant genes Nrf2, NQO-1, and HO-1, decreased expression of Keap1, and reduced inflammation through decreased expression of IL-6, IL-1β, and TNF-α [43]. The aqueous extract of MBSC also exhibits anti-inflammatory activity in lipopolysaccharide (LPS)-induced RAW 264.7 macrophages [44]. The results in this study showed that treatment of HG-exposed HaCaT cells and wounds with EE, AE, LMW, and CP could reduce intracellular ROS, improve cell proliferation, and enhance cell migration, suggesting that the extracts might enhance antioxidant response mechanisms through Nrf2 pathway activation, exert anti-inflammatory effects, and regulate collagen balance in diabetic wound healing. Although EE, AE, LMW, and CP exhibited ROS-suppressing activity, only EE and LMW showed a promising effect of decreasing ROS accumulation while enhancing cell proliferation and migration. However, EE was chosen for further elucidation of gene expression in HG-exposed HaCaT wounds, since EE demonstrated the highest potency in enhancing wound closure and was obtained in higher yield using a simple extraction method. In contrast, LMW required more preparation steps and yielded less.

Nrf2 is involved in anti-inflammatory and antioxidant stress activities. It is highly expressed in the skin and plays a critical role in diabetic wound healing. In a hyperglycemic state, oxidative stress and inflammation occur, leading to Nrf2 suppression and delaying diabetic wound healing. Therefore, Nrf2 contributes to diabetic wound healing by alleviating oxidative stress, promoting proliferation and migration, reducing apoptosis, enhancing transforming growth factor-β1 (TGF-β1) expression, and suppressing matrix metalloproteinase 9 (MMP-9) [14]. In this study, Nrf2 downregulation in HG-exposed HaCaT wounds was observed (Figure 8A), consistent with the increased intracellular ROS (Figure 6), which led to inflammation, as shown by TNF-α upregulation (Figure 8F). The upregulation of MMP-9 and the downregulation of TGF-β1, which resulted in the suppression of cell migration, also corresponded to the lowest percentage of wound closure (Figure 7) in the untreated HG-exposed wound.

EE treatment of the HG-exposed HaCaT wound upregulated Nrf2 and increased the expression of the downstream genes NQO-1, CAT, and SOD2, indicating a promising function of EE as an Nrf2 activator, which was supported by reduced intracellular ROS and increased cell proliferation (Figure 6) in HG-exposed HaCaT cells and enhanced cell migration in HG-exposed HaCaT wounds (Figure 7). There is a positive relationship between wound-healing rate and the expression levels of Nrf2 and HO-1 in diabetic mice [45]. EE treatment of HG-exposed wounds also promoted upregulation of HO-1, SOD, and CAT genes, which are downstream of the Nrf2 pathway and help destroy ROS. Nrf2 could also inhibit cell apoptosis, stimulate TGF-β1 expression, and attenuate MMP-9 expression in diabetic wound healing in keratinocytes [14], which could be achieved through the upregulation of TGF-β1 and downregulation of MMP-9 by EE treatment.

The substances or herbal extracts that enhance the function of Nrf2 are therefore considered to have potential diabetic wound-healing effects. The activation of Nrf2 in keratinocytes is crucial for promoting diabetic wound healing by mitigating oxidative stress, promoting proliferation and migration, reducing apoptosis, enhancing TGF-β1 expression, and suppressing MMP-9 [14]. Hence, EE, which exerted hypoglycemic effects and acted as an Nrf2 activator, could be considered a high-potential extract for the prevention of diabetic wounds and the mitigation of diabetic complications.

Under high-glucose conditions, the Nrf2 pathway was impaired, but the gene expression levels of NQO-1, HO-1, CAT, SOD2, and TGF-β1 in the untreated HG-exposed HaCaT wounds were not different from those in the untreated NG-exposed HaCaT wounds (Figure 8). This may be because the 24 h high-glucose exposure in the HG-exposed HaCaT wound was non-chronic and insufficient to detect differences in gene expression between the NG- and HG-exposed wounds. However, treatment with EE in HG-exposed HaCaT wounds increased the gene expression of Nrf2 and the downstream antioxidant genes NQO-1, CAT, and SOD2, suggesting that EE functions as an Nrf2 activator. However, EE did not alter HO-1 expression levels, suggesting that it may not act via HO-1. Therefore, enzyme activity assays for CAT and SOD, Western blotting for protein detection, or in vivo treatment would be required.

In addition to the Nrf2 pathway, delayed wound healing also relates to the increase in MMPs and the decrease in TGF-β1 in the keratinocytes of diabetic wounds. TGF-β1 is a cytokine that promotes re-epithelialization and enhances keratinocyte migration for wound closure. The upregulation of TGF-β1 enhances diabetic wound healing [46]. Although keratinocyte migration is assisted by ECM degradation by MMPs, excessive MMP activity delays wound healing in a murine model [47]. MMP-9 functions as a gelatinase and retards the re-epithelialization stage of wound healing. MMP-9 inhibitors have emerged as targets for diabetic wound treatment [48]. Diabetic wounds are also caused by chronic inflammation and the prolongation in this inflammatory phase, retard wound healing. Hence, decreased expression of cytokines, such as TNF-α and IL-6, reflecting an anti-inflammatory effect [3,11], is essential for wound healing. The downregulation of pro-inflammatory cytokines, including TNF-α and IL-6, in LPS-induced macrophages (J774) by the fraction consisting of vitexin and isovitexin from mung bean seed [49] suggests the anti-inflammatory effects of extracts containing vitexin and isovitexin. Hence, the decrease in TNF-α expression following EE treatment might suggest the anti-inflammatory potential of EE.

Berberine, a positive control in this study, is a ROS inhibitor via the Nrf2 pathway [50] and is now being developed as a diabetic wound-healing product. The scratch assay and intracellular ROS assay showed that it did not induce cell migration to close the wound (Figure 7) or restore cell viability after ROS formation (Figure 6), which corresponded to its function of inhibiting cell proliferation and migration by inhibiting CDC6 expression and interfering with the JAK-STAT3 signaling pathway in HaCaT cells, supporting its potential use in psoriasis treatment [51]. Topical treatment with berberine in streptozotocin (STZ)-induced diabetic rats accelerates wound healing by inhibiting oxidative damage, attenuating inflammation, and promoting collagen synthesis [52].

In summary, the results indicated the potential of EE for high-glucose wound healing through its glucose-lowering effect, which promotes glucose uptake and inhibits α-glucosidase function. EE mitigated oxidative stress through the Nrf2 pathway. The effects of EE that downregulated MMP-9 suggests suppression of collagen degradation, and the upregulation of TGF-β1 indicates enhanced cell migration for wound closure. Pretreatment with an Nrf2 activator promotes diabetic wound healing by reducing oxidative stress [11]. EE, which was found to be a promising Nrf2 activator, might be effective in delaying the progression of diabetic ulcers at earlier stages or in the pre-ulcer stage, when the symptoms of inflammation and delayed wound healing start. The results demonstrate the potential for EE to serve as a food supplement to combat ROS and inhibit glycation in hyperglycemic conditions, as well as a topical product to prevent or delay the progression of diabetic wounds. This finding supports the zero-waste concept by using MBSC, an industrial byproduct of vermicelli, to produce high-value health-benefit products.

However, this study has some limitations. The effects of MBSC extracts and EE were evaluated only in vitro; therefore, in vivo assays should be conducted in future studies. Toxicity testing of EE should be performed to ensure safe use. A more molecular-level approach to pathway elucidation should be explored to elucidate the mechanism of diabetic wound healing in detail.

## 5. Conclusions

This study highlights the ability of mung bean seed coat extracts to enhance diabetic ulcer healing through multiple mechanisms, including antioxidant, hypoglycemic, AGE-inhibitory, Nrf2-activating, anti-inflammatory, and wound-healing effects, including increased cell migration and regulation of ECM balance. Among the MBSC extracts evaluated, the EE, which was rich in vitexin and isovitexin, exhibited superior activity. These findings align with the zero-waste policy of utilizing MBSC, a byproduct of the mung bean industry, to develop products that delay diabetic wound progression by addressing the risk factors at their source.

## Figures and Tables

**Table 1:** Primers used in this study.

Gene	Forward Primer (5′ → 3′)	Reverse Primer (5′ → 3′)	Ref.
Nrf-2	TCAGCGACGGAAAGAGTATGA	CCACTGGTTTCTGACTGGATGT	[28]
HO-1	TCTCTTGGCTGGCTTCCTTA	TAGGCTCCTTCCTCCTTTCC	[29]
NQO-1	GAAGAGCACTGATCTACTGGC	GGATACTGAAAGTTCGCAGGG	[30] *^,a^
TGF-β1	GTGGAAACCCACAACGAAAT	CGGAGCTCTGATGTGTTGAA	[29]
SOD2	GGAAGCCATCAAACGTGACTT	CCCGTTCCTTATTGAAACCAAGC	[30] *^,b^
CAT	TGGGATCTCGTTGGAAATAACAC	TCAGGACGTAGGCTCCAGAAG	[30] *^,c^
TNF-α	GCTGCACTTTGGAGTGATCG	TCACTCGGGGTTCGAGAAGA	- **
MMP-9	ACGATGACGAGTTGTGGTCC	GGTTTCCCATCAGCATTGCC	[31]
β-actin	CCTGGCACCCAGCACAAT	GGGCCGGACTCGTCATAC	[28]

* Primers were from Primers Bank, with the primer bank ID as follows—a: 70995395c1; b: 67782304c2; c: 260436906c3, respectively; ** the primers were designed in this study from the mRNA accession no. NM_000594.4.

**Table 2:** Mung bean seed coat extracts and biomarker contents.

Extract	Weight (g)	Yield (*w*/*w*)	Vitexin (*w*/*w*)	Isovitexin (*w*/*w*)
80% EtOH extract (EE)	9.05	11.31% of DW	6.2% of EE	14.4% of EE
Aqueous extract (AE)	6.19	7.74% of DW	0.59% of AE	1.17% of AE
-Low Molecular weight (LMW)	1.32	1.64% of DW 21.25% of AE	1.35% of AE 6.3% of LMW	3.56% of AE 16.7% of LMW
-Crude polysaccharide (CP)	0.83	1.04% of DW 13.42% of AE	0.006% of AE 0.045% of CP	0.015% of AE 0.109% of CP

DW = mung bean seed coat dry weight.

**Table 3:** Total phytochemical contents of the extracts.

Extract	TPC (mg GAE/g Extract)	TFC (mg QE/g Extract)	Total Amino Acid (mg GE/g Extract)	Total Polysaccharide (mg ME/g Extract)
80% EtOH extract (EE)	23.71 ± 1.61	23.47 ± 1.83	5.85 ± 1.08	n.d.
Aqueous extract (AE)	27.44 ± 0.30	12.06 ± 0.28	16.61 ± 5.91	335.33 ± 13.27
Low Molecular weight (LMW)	48.07 ± 0.74	24.67 ± 2.87	10.612 ± 2.10	n.d.
Crude polysaccharide (CP)	20.53 ± 0.72	7.25 ± 0.19	4.71 ± 0.00	432.47 ± 18.42

n.d. = not determined.

**Table 4:** Antioxidant and α-glucosidase inhibitory activities of the extracts.

Samples	FRAP (mg Trolox/g Extract)	IC_50_ (µg/mL)	
		ABTS	α-Glucosidase Inhibition
80% EtOH extract (EE)	39.05 ± 1.70	353.20 ± 18.85	182.30 ± 4.45
Aqueous extract (AE)	42.31 ± 1.12	267.77 ± 17.12	226.53 ± 6.06
Low Molecular weight (LMW)	71.58 ± 5.06	184.23 ± 10.90	223.42 ± 7.51
Crude polysaccharide (CP)	40.27 ± 1.36	407.28 ± 44.79	164.59 ± 4.57
Trolox	-	7.67 ± 0.13	n.d.
Acarbose	n.d.	n.d.	78.69 ± 0.55

n.d. = not determined.

## Data Availability

The original contributions presented in this study are included in the article and the Supplementary Material. Further inquiries can be directed to the corresponding author.

## Supplementary Materials

The following supporting information can be downloaded at: https://www.mdpi.com/article/10.3390/antiox15091119/s1, Table S1: Effect of mung bean seed coat extracts (EE, AE, LMW, and CP) on wound area (µm^2^) and % wound closure of the high glucose-exposed HaCaT wounds; Figure S1: % Wound closure of HG-exposed HaCaT wound by 100 µg/mL MBSC extracts at 48 h.

## Abbreviations

The following abbreviations are used in this manuscript:

MBSC	Mung bean seed coat
BSA	Bovine serum albumin
HG	High glucose
ROS	Reactive oxygen species
Nrf2	Nuclear factor erythroid 2–related factor 2
NQO-1	NAD(P)H) quinone oxidoreductase-1
SOD2	Superoxide dismutase
CAT	Catalase
TNF-α	Tumor necrosis factor-α
TGF-β1	Transforming growth factor-β1
MMP-9	Matrix metalloprotease-9
AGEs	Advanced glycation end-products
ECM	Extracellular matrix
IL	Interleukin
MGO	Methylglyoxal
Keap1	Kelch-like ECH-associated protein 1
HO-1	Heme oxygenase-1
AREs	Antioxidant response elements
JAK-STAT3	Janus Kinase and Signal Transducer and Activator of Transcription 3
