# Supplementary material for: Mung Bean Seed Coat Extract Promotes Diabetic Wound Healing in High-Glucose-Exposed HaCaT Keratinocytes
Source: Antioxidants (Basel). 2026 Sep 4;15(9):1119. doi: 10.3390/antiox15091119 (PMC13603269; doi:10.3390/antiox15091119)
Supplement: Supplementary file 1 [file antioxidants-15-01119-s001.zip › antioxidants-4440263-supplementary.pdf]

Supplementary Materials

Mung Bean Seed Coat Extract Promotes Diabetic Wound Healing in High-Glucose-Exposed HaCaT Keratinocytes

Sineenad Teerapatpaisan [1], Alisa Naladta [2], Kamonpan Wanichsri [1], Penpimol Ponchunchoovong [1], Suthasinee Thapphasaraphong [1] and Natsajee Nualkaew [1,*]

[1] Faculty of Pharmaceutical Sciences, Khon Kaen University, Khon Kaen 40002, Thailand; sineenad.te@kkumail.com (S.T.); kamonpan.w@kkumail.com (K.W.); penpimol.p@kkumail.com (P.P.); sutpit1@kku.ac.th (S.T.)

[2] Faculty of Sciences, Khon Kaen University, Khon Kaen 40002, Thailand; alisa_n@kkumail.com

* Correspondence: nnatsa@kku.ac.th

Supplementary Table

Table S1: Effect of mung bean seed coat extracts (EE, AE, LMW, and CP) on wound area (μm²) and % wound closure of the high glucose-exposed HaCaT wounds.

Treatment	Wound area (μm²)					% wound closure*			
	0 h	12 h	24 h	36 h	48 h	12 h	24 h	36 h	48 h
HG (40 mM glucose)	280167±42609	223207±32865	211897±30631	204900±27811	173720±32116	20.3±1.4 [a]	24.3±2.0 [b]	26.7±1.4 [b]	38.2±2.4 [c]
NG (25 mM glucose)	262517±26975	193294±26728	156990±22824	136529±36979	101718±20635	26.5±2.5 [a]	40.4±2.6 [b]	48.6±8.4 [b]	61.5±4.2 [c]
25 μM Berberine	458822±383234	331465±263241	286490±219046	278061±216619	260996±186454	26.1±5.4 [a]	35.1±5.4 [a]	37.5±7.6 [a]	39.3±7.8 [a]
5% FBS	281740±31457	197871±24548	21510±5237	0±0	0±0	29.8±1.0 [a]	92.4±1.1 [b]	100.0±0.0 [c]	100.0±0.0 [c]
LMW 50 μg/mL	289462±37351	227122±41880	215118±50932	187238±46018	152399±33626	21.9±4.8 [a]	26.4±8.9 [a]	35.9±8.9 [a,b]	47.3±9.3 [b]
LMW 100 μg/mL	223321±12767	169548±9624	134973±7150	134268±14912	118598±5186	23.9±7.0 [a]	39.5±1.4 [b]	39.8±6.8 [b]	46.7±5.5 [b]
EE 50 μg/mL	267333±30231	206108±28791	186316±29485	162253±21453	142819±23326	23.0±2.9 [a]	30.5±3.8 [a,b]	39.3±4.8 [b,c]	46.7±4.3 [c]
EE 100 μg/mL	295971±35984	210860±26982	159539±25545	140690±18601	121391±17019	28.8±0.7 [a]	46.3±2.1 [b]	52.5±2.1 [c]	59.0±1.0 [d]
AE 50 μg/mL	285093±44490	228323±48240	210544±40409	194919±30227	185820±26468	20.4±5.0 [a]	26.4±3.0 [a,b]	31.6±1.5 [b,c]	34.7±1.0 [c]
AE 100 μg/mL	239971±22249	188039±19227	172259±17581	151745±13836	139671±8986	21.7±1.3 [a]	28.2±4.5 [a]	36.7±2.0 [b]	41.6±3.7 [b]
CPE 50 μg/mL	266119±4886	217236±5682	197271±11255	189916±4738	178484±10657	18.4±1.2 [a]	25.9±3.0 [b]	28.6±1.0 [b,c]	33.0±3.2 [c]
CPE 100 μg/mL	281877±13333	221889±18012	192535±19626	185303±15709	171868±19286	22.7±1.7 [a]	33.7±1.9 [b]	36.6±2.3 [b]	41.5±1.3 [c]

Data are presented as mean ± S.D., *% wound closure was statistically compared within the same group. Different letters indicate significant differences % wound healing between exposure time in each treatment at $p < 0.05$ by Tukey's multiple comparison test ($n = 3$).

Supplementary Figure

Figure S1. % Wound closure of HG-exposed HaCaT wound by 100 µg/mL MBSC extracts at 48 h

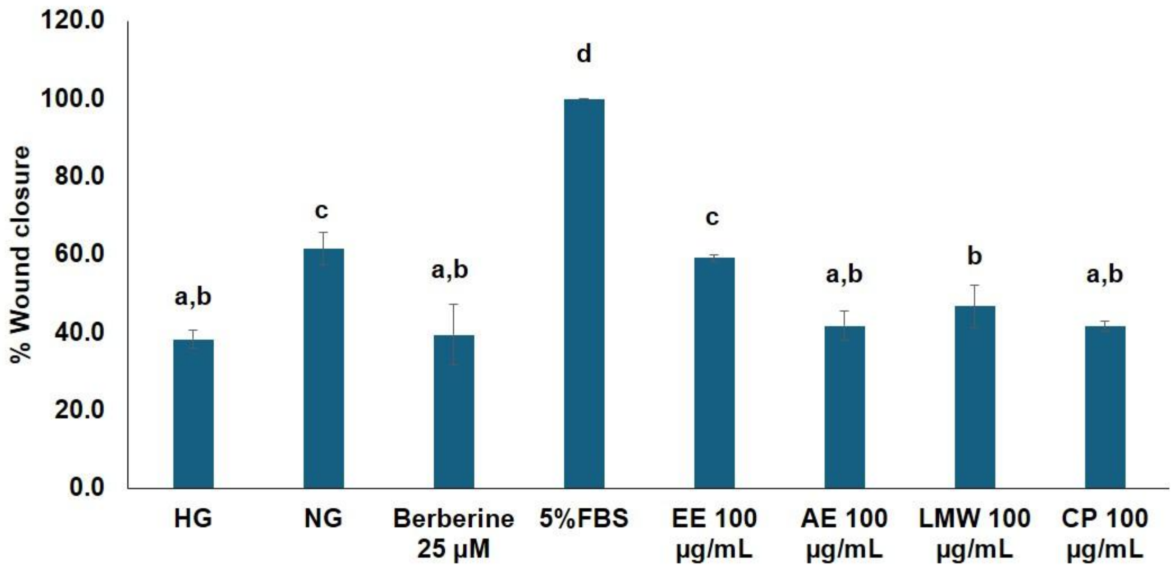

Figure S1. % Wound closure of HG-exposed HaCaT wound by 100 µg/mL MBSC extracts at 48 h:

(HG: untreated-high glucose-exposed HaCaT wound; NG: untreated normal-glucose-exposed HaCaT wound. Data are expressed as the mean ± SD. *$P < 0.05$, different letters indicate significant differences between groups at $p < 0.05$ by Tukey's multiple comparison test ($n = 3$).
